# Rainfall Intensity and Quantity Estimation Method Based on Gamma-Dose Rate Monitoring

**DOI:** 10.3390/s21196411

**Published:** 2021-09-25

**Authors:** Valentina Yakovleva, Grigorii Yakovlev, Roman Parovik, Aleksey Zelinskiy, Aleksey Kobzev

**Affiliations:** 1 Nuclear Fuel Cycle Department, School of Nuclear Science & Engineering, Tomsk Polytechnic University, 634050 Tomsk, Russia; vsyakovleva@tpu.ru (V.Y.); azelinskiys@yandex.ru (A.Z.); 2 Institute of Applied Mathematics and Computer Science, Tomsk State University, 634050 Tomsk, Russia; yakovlev-grisha@mail.ru; 3 Institute of Cosmophysical Research and Radio Wave Propagation, Far Eastern Branch of the Russian Academy of Sciences, 684034 Kamchatskiy Kray, Russia; 4 Institute of Monitoring of Climatic and Ecological Systems of Siberian Branch of the Russian Academy of Sciences, 634055 Tomsk, Russia; kaa@imces.ru

**Keywords:** rainfall intensity, γ-dose rate, γ-background, rain sensor, method, radon decay product, liquid precipitation, atmosphere, activity, simulation

## Abstract

The features of the atmospheric γ-background reaction to liquid atmospheric precipitation in the form of bursts is investigated, and various forms of them are analyzed. A method is described for interpreting forms of the measured γ-background response with the determination of the beginning and ending time of precipitation, the distinctive features of changes in the intensity of precipitation and the number of single (separate) events that form one burst. It is revealed that a change in the intensity of precipitation in one event leads to a change in the γ-radiation dose rate increase speed (time derivative). A method of estimating the average value of the intensity and amount of precipitation for one event, reconstructing the intensity spectrum from experimental data on the dynamics of the measured dose rate of γ-radiation, is developed. The method takes into account the radioactive decay of radon daughter products in the atmosphere and on the soil surface during precipitation, as well as the purification of the atmosphere from radionuclides. Recommendations are given for using the developed method to correct for changes (daily variations) in radon flux density from the ground surface, which lead to variations in radon in the atmosphere. Experimental verification of the method shows good agreement between the values of the intensity of liquid atmospheric precipitation, calculated and measured with the help of shuttle and optical rain precipitation gauges.

## 1. Introduction

Systematic monitoring of precipitation is carried out by specialized bodies and national meteorological centers. They perform measurements and observations at specified standard times and locations; usually, they monitor total rainfall over 12 h. The current pace and magnitude of climate change is shaping new trends in the environment state. To identify and control them, it is necessary to have a developed network of stations for monitoring geophysical characteristics, covering large areas with high spatial resolution [1,2].

At the same time, all around the world, with the growing demand for carbon-free energy, there has been a revision in the attitude toward nuclear energy (nuclear power industry). Due to the growth in the number of nuclear power plants and the stricter requirements for nuclear and radiation safety, the number of stations for the monitoring of background radiation is growing [2,3,4].

It has been repeatedly reported [5,6,7,8,9,10,11,12,13] that periods of precipitation are accompanied by an abnormal rapid increase (bursts) of the γ-radiation background. This phenomenon is explained by the processes of washing out short-lived β- and γ-emitting decay products of radon and thoron from the atmosphere onto various surfaces and is known as “radon washout” [14].

Much has already been done to determine the relationship between the γ-background and precipitation intensity. Attempts to find a quantitative relationship between the intensity of precipitation and the magnitude of bursts in the dose rate of γ-radiation were undertaken earlier in [5,6,7], but no significant relationship was found. Many models have been developed to analyze bursts of ambient dose rate of γ radiation associated with precipitated radon decay products, taking into account various dynamic and physical processes, having different levels of complexity and based on various assumptions [7,8,9,10,11]. For example, the “rainout-washout” model developed in [7], which divides the atmosphere into two parts—“in the cloud” and “under the cloud”—has not yet received experimental confirmation.

Nevertheless, in the work [11], a mathematical model was proposed that allows to restore the dose rate of γ radiation during the fallout of liquid atmospheric precipitation. Its effectiveness is confirmed by a high determination coefficient (R${}^{2}$ = 0.81–0.99) between the measured and recreated ambient dose equivalent rate during single and multiple rain events. In this work, only washout from the subcloud space was considered, while the error between the measured and reconstructed dose rate was rather small (RMSE = 0.0045 μSv·h${}^{1}$). The results of this work confirm that the radionuclides are washed out mostly from the subcloud space, and the contribution of radionuclides that are in the cloud is likely negligible.

Estimation of the intensity and other precipitation characteristics by dynamics of the γ-radiation dose rate is quite a difficult task. Numerous studies have shown that knowledge of the γ-radiation dose rate alone is not sufficient. In [12], this was due to insufficiently high temporal resolution of the data. In works [9,10,15], the authors found that it is necessary to take into account the washout ability of precipitation, as it depends on its duration and intensity. In [16], the absence of a significant relationship between the intensity of precipitation and the γ-radiation dose rate spike was explained by the fact that during prolonged precipitation that lasts many hours, the atmosphere gradually clears, and hence, the amount of deposited radon decay products decreases. Therefore, the next event results in the deposition of fewer radon decay products.

Considering the above, the aim of our work was to develop a simple method of estimating the average values of intensity and amount of precipitation for one event (as well as intensity spectrum) based on the dynamics of the measured γ-radiation dose rate. For this purpose, it was necessary to solve a number of tasks: (1) to study the features of the atmospheric γ-background response to liquid precipitation; (2) to develop a method for determining the average per-event values of the intensity and amount of precipitation (as well as spectrum) from the experimental data on the γ-radiation dose rate; and (3) to perform experimental verification of the method.

Section 2 describes the experimental equipment used for method testing. Section 3 considers the classification of the γ-background responses to liquid atmospheric precipitation, and describes the characteristic features of rain cases in the γ monitoring data. Section 4 describes the method called GammaRain, which takes into account the radioactive decay of radon decay products in the atmosphere and on the ground surface, atmospheric clearance from radionuclides during precipitation, and changes in radon flux density from the ground surface. The last two sections present the results of the experimental verification of the method and the general conclusion.

## 2. Experiment Equipment

Each year, starting from 2017, from the moment of snow melting and until the beginning of snow cover establishment, the measurements of γ-radiation dose rate ambient equivalent were performed with high data sampling rate of 1 minute, using scintillation detector BDKG-03 (made by ATOMTECH, Republic of Belarus). Detector BDKG-03 as a sensitive element contains NaI(Tl) scintillator with dimensions Ø25 × 40 mm. The range of detectable energies of gamma radiation is from 50 keV to 3 MeV. The choice of the γ-background sampling rate of 1 minute is due to the statistical error, which is 4–10% in the absence of precipitation, and during the period of precipitation, is reduced by increasing the statistics to 2–4%. Reducing the data sampling rate to less than 1 min for this type of gamma detector will increase the statistical uncertainty, but increasing the data sampling rate to 2–3 min or more is not acceptable for intensity spectrum reconstruction purposes because the precipitation process is classified as rapidly changing. BDKG-03 detectors were installed at the experimental site of the Geophysical Observatory of the Institute of Monitoring of Climate and Ecological Systems of Siberian Branch of the Russian Academy of Sciences (IMCES SB RAS) at the height of 1 m from the ground surface. The choice of γ-background measurement height equal to 1 m from the soil surface is conditioned by the requirements for the control of the radiation situation for the population. Same installation height provides comparability of measurement results for different territories both in Russia and abroad.

Precipitation intensity data with high temporal resolution were recorded by a Davis Rain Collector II shuttle precipitation gauge (Davis Instruments, Hayward, CA, USA). An optical (laser) precipitation gauge, OPTIOS, developed at IMCES SB RAS, was installed at a distance of not more than 10 m from the γ-radiation detector.

To measure the radon flux density from the soil surface, we used an EQF-3200 radiometer (SARAD, Germany) with an accumulation chamber as well as a measuring complex “Alfarad plus—AR” (NTM-Zashchita LLC, Moscow, Russia).

## 3. γ-Background Response to Liquid Atmospheric Precipitation

**Figure 1:**
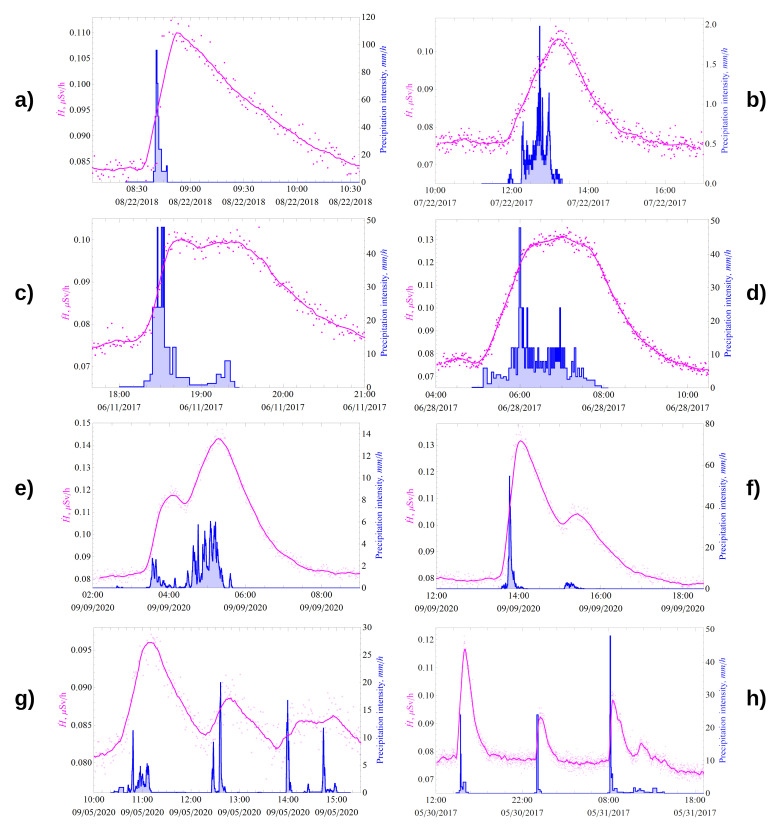
Different forms of γ-background responses to liquid atmospheric precipitation (occurred at IMCES, Tomsk, RF): (**a**) peak with left position of maximum; (**b**) middle maximum position; (**c**) flat/plateau shaped; (**d**) bell-shaped; (**e**,**f**) double-humped shape; (**g**) wave-shaped; (**h**) toothed shape.

**Figure 2:**
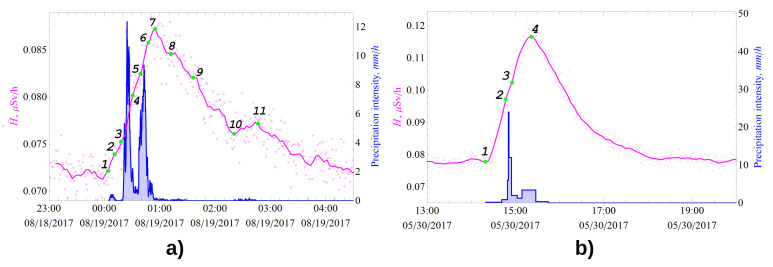
Analysis of γ-background dynamics during type III precipitation of (**a**) 18 August (**b**) 30 May (occurred at IMCES, Tomsk, RF).

Detailed analysis of the results of long-term experimental data showed that all registered bursts in the γ-background, which have no definite periodicity, are caused by precipitation (excluding bursts caused by errors in operation of γ-radiation detectors). Figure 1 and Figure 2 show the dynamics of the γ-dose rate (pink) and precipitation intensity (blue). Minute data are indicated by dots, and the solid line shows data smoothed by a moving average of 5–7 points. During precipitation, the greater the growth of the γ-radiation dose rate, the greater the number of registered pulses and, correspondingly, the smaller the total uncertainty of measurement results, which is well illustrated by Figure 1d,e and Figure 2b. Analysis of experimental data (Figure 1) allowed to reveal that the γ-background outburst value does not correlate with the precipitation intensity, which agrees well with the experimental data on the γ-background [5,6,10].

The response of the γ-radiation dose rate to precipitation, manifested as anomalous bursts in the γ-background, was studied in detail, and their classification was made (Figure 1). Four characteristic forms of bursts in the γ-background, corresponding to liquid atmospheric precipitation with different parameters, were distinguished:

(I) A peak with different position of the maximum: on the left (Figure 1a); in the middle (Figure 1b) or on the right;
(II) Flat/plateau (as it stays on about same level after reaching max value/bending point) (Figure 1c) and bell (Figure 1d);
(III) Double-humped (Figure 1e,f);
(IV) Wavy/wave-shaped (Figure 1g) and toothed/saw-toothed/jagged (Figure 1h).

The characteristic form of the burst, which can be called “classic”, for single short- to medium-intensity rains is shown in Figure 1a. The rain shower that occurred on 22 August 2018 (at IMCES, Tomsk, Russian Federation) had an intensity of more than 100 mm/h at its maximum. The responses of the classical form are characterized by a sharp increase and, after reaching the maximum, by an exponential decrease in accordance with the law of radioactive decay ($e^{-\lambda t}$) with exponent power in the range of λ≈ 2.8–4.3 $\times 10^{-4}$s${}^{-1}$; the last value is close to the decay constant for ${}^{214}$Pb.

The explanation for the increase in γ-radiation dose rate during precipitation periods is that the γ-emitting short-lived radon decay products ${}^{214}$Pb and ${}^{214}$Bi are washed out of the atmosphere by precipitation onto the soil surface. Other natural radionuclides, for example, γ-emitting decay products of thoron, practically do not influence the value of γ-radiation dose rate because their activity in the near ground atmosphere is much less than the activity of ${}^{214}$Pb and ${}^{214}$Bi. ${}^{218}$Po, the 1st decay product of ${}^{222}$Rn, is α-active, so it does not influence the γ-background.

Analysis of long-term data on γ-background responses to liquid atmospheric precipitation has allowed us to develop a method for interpreting various forms of measured γ-background response by characteristic features of changes in the precipitation intensity:

- The time of the beginning of γ-background growth corresponds to the beginning of precipitation (point 1 on Figure 2);
- The growth rate of γ-radiation ambient equivalent dose rate (change of slope angle of growth curve or dose rate derivative characterizes current value of precipitation intensity);
- The time of the maximum onset (if there are several in one burst, the 1st one) corresponds to the end of precipitation (except for the type II forms), corresponding to point 7 on Figure 2;
- The exponential decrease in the γ-background after the maximum means that the radioactive decay of ${}^{214}$Bi and ${}^{214}$Pb radon decay products deposited on the ground has begun, so after about 3 h, their activity decreases by more than 2 orders of magnitude;
- If after reaching the maximum we observe a flat (small dip and continued growth) or bell shape, this clearly indicates that the rain continues with a variable low intensity with respect to the previous interval, which is well illustrated in Figure 1d;
- If we can detect a weak decrease in the γ-background after reaching the maximum, as shown in Figure 1c, but there are no clearly marked 2nd and subsequent maximums, this means that almost immediately after the 1st, the 2nd rain began (the rain did not end but continued with a lower intensity, which is still considered the same event);
- If the subsequent precipitation events start before ${}^{214}$Bi and ${}^{214}$Pb have completely decayed, we will see 2 or more maxima in the γ-background response, depending on the number of events and precipitation characteristics (intensity, amount, duration, etc.), as seen in Figure 1;
- Precipitation lasting for about half an hour usually results in “sharp” peaks in the γ-background;
- Double-humped or wave-shaped response forms are caused by two or more consecutive precipitation events. The time between the end of the previous event and the beginning of the next one is shorter than the time of radioactive equilibrium restoration between radon and its daughter decay products in the atmosphere (less than 3 h);
- The toothed form is characteristic for series of short duration rain showers with a periodicity of more than 3 h.

High- and moderate-intensity rains usually have an average duration of 15 min, but not more than 30 min, and lead to a response in the γ-background with a shape close to the “classical” (Figure 1a and Figure 2). If the duration of rainfall is more than 1 h, the average intensity of such rain is usually less than 10 mm/h.

Let us consider in detail how to determine the start and end times of precipitation as well as the number of bursts in the intensity spectrum forming a single event. The excess of γ-radiation dose rate over the background value at the maximum is defined as the value of $\dot{H}$ at point 7 minus $\dot{H}$ at point 1 (Figure 2a) or the value of $\dot{H}$ at point 4 minus point 1 (Figure 2b). The current value and the change in the intensity of rain is determined by the derivative $\frac{d\dot{H}\left(t\right)}{dt}$.

The beginning of the rain on 19 August (Figure 2a) is accompanied by an increase in γ-background (segment 1–2 in Figure 2a), and then the intensity decreases, which leads to a change in the slope angle (decrease in the derivative), which is shown in segment 2–3. Then, the derivative increases (segment 3–4), which corresponds to the 2nd peak of rain. Segment 4–5 shows a decrease in intensity as the slope angle decreases in this plot. Segment 5–6 corresponds to the 3rd increase in intensity of this rain event. After point 6, the intensity begins to decrease; point 7 can be considered the time of the rain’s end, which is followed by the radioactive decay of ${}^{214}$Pb and ${}^{214}$Bi, according to the exponential law. Small drizzling precipitation contributes perturbations to the exponential decrease in the γ-background decay (segments 8–9 and 10–11 in Figure 2a).

To determine the average precipitation intensity per event I(t), we take segments 1–7.

The higher the intensity of precipitation, the easier it is to interpret the spectrum of its intensity, which is well seen in Figure 2b. By analogy, we analyze the case in Figure 2b. Point 1 corresponds to the beginning of precipitation, while point 4 corresponds to the end of precipitation. Points 2 and 3 are the change in intensity. The dynamics of the derivative $\frac{d\dot{H}\left(t\right)}{dt}$ is discussed in detail in Section 4.3 and later.

As a result, long-term experiment on investigation of influence of heavy precipitation on surface atmosphere radiation background allowed to reveal that current precipitation intensity I(t) is determined exactly by the increase in speed of the γ-radiation dose rate growth, which is characterized by derivative $\frac{d\dot{H}\left(t\right)}{dt}$. The obtained analysis results formed the basis for the GammaRain method of precipitation intensity and amount determination by atmospheric γ-background.

## 4. GammaRain Method for Determining the Intensity and Amount of Precipitation by Atmospheric γ-Background

In this work, only the process of washout of daughter radon decay products by precipitation “from under the cloud” is considered. We also assume that the γ-radiation dose rate burst is caused by γ-radiation of ${}^{214}$Pb and ${}^{214}$Bi, short-lived daughter radon decay products deposited on the ground surface, as the main contributors to the total dose rate, compared to the rest of the radon and thoron decay products.

**Figure 3:**
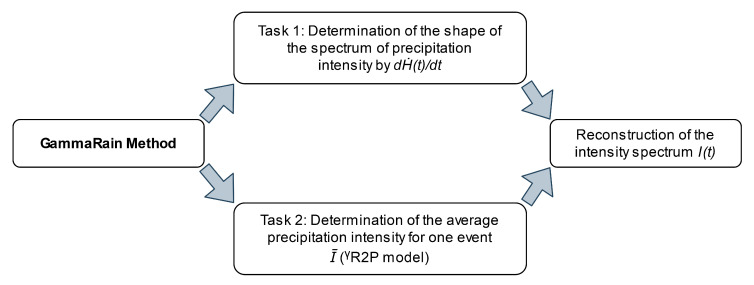
Algorithm (scheme) of GammaRain method implementation.

The method for estimating the mean values of the amount and intensity of liquid atmospheric precipitation per event was developed based on a set of values that we can realistically measure or estimate from known geophysical data and nuclear constants. Apart from the measured dose rate of γ radiation, everyone can measure radon flux density from the soil surface or estimate it on the basis of ${}^{226}$Ra content in the soil, according to known models. Figure 3 shows an algorithm (scheme) of GammaRain method implementation to determine the intensity (time spectrum) and amount of precipitation on the atmospheric γ-radiation background, which is divided into two separate tasks. Task 1 is to determine the form of the precipitation intensity spectrum by calculating the time derivative $\frac{d\dot{H}\left(t\right)}{dt}$ with a given step, using γ-background monitoring data (described in detail in Section 4.3). As studies have shown, the derivative $\frac{d\dot{H}\left(t\right)}{dt}$ determines the form of the precipitation intensity spectrum. Task 2 is to determine the average value of rain intensity for one event; for this purpose, the model ^γ^R2P described in Section 4.1 was developed. It is possible to reconstruct the spectrum of rain intensity only when we know both the average intensity and the γ radiation dose rate derivative spectrum. The spectrum reconstruction is performed by equating the area under the curve $\frac{d\dot{H}\left(t\right)}{dt}$ for the delta t interval to the calculated value of the average precipitation amount (or using average intensity) and determining the constant by which the result is multiplied to obtain the correct total precipitation amount.

### 4.1. ^γ^R2P Model to Determine the Average Per-Event Intensity of Precipitation

The estimation of average values of intensity and amount of precipitation per one event is made by monitoring the data of the γ-radiation dose rate. For this purpose, let us write down the initial equality as follows:

(1) $$\Delta {\dot{H}}_{\mathrm{measured}}=\Delta {\dot{H}}_{\mathrm{estimated}}$$

where $\Delta {\dot{H}}_{\mathrm{measured}}$ and $\Delta {\dot{H}}_{\mathrm{estimated}}$ are the values of the burst (excess over the background value at the maximum) of γ-radiation dose rate measured and calculated, respectively, μSv/h.

The value $\Delta {\dot{H}}_{\mathrm{measured}}$ can be determined from experimental data as follows:

(2) $$\Delta {\dot{H}}_{\mathrm{measured}}={\dot{H}}_{\mathrm{end}}-{\dot{H}}_{0},$$

where ${\dot{H}}_{0}$ is value of γ-radiation dose rate at the moment *t*_0_ corresponding to the beginning of liquid atmospheric precipitation, which is defined as a point after which continuous growth of dose rate is observed during time *t*_end_ up to maximum value ${\dot{H}}_{\mathrm{end}}$, μSv/h (according to Figure 2); ${\dot{H}}_{\mathrm{end}}$ is maximum value of the γ-radiation dose rate in “burst”, μSv/h (according to Figure 2).

The analysis of both the experimental data and theoretical material from the field of nuclear physics and interaction of ionizing radiation with the substance allows to assert that the value of the γ-radiation dose rate burst $\Delta \dot{H}$, μSv/h is proportional to the deposited on the soil surface radionuclide activity at the end of the precipitation; additionally, each *j*-th radionuclide of unit activity makes a constant contribution ${\dot{H}}_{j}^{1Bq}$ to the total dose rate of γ-radiation of the surface atmosphere at a unit distance from the soil surface, which depends on the nuclear and physical characteristics of the radionuclide.

If the activity of the radionuclides deposited on the soil surface by rainfall $A_{j}^{s}\left(t=t_{\mathrm{end}}\right)$, Bq/m^2^ is known, at the precipitation end time *t*_end_ and the dose coefficients at unit activity for these radionuclides, we can write down the exact equality between the measured value of the γ-radiation dose rate burst (excess) and the activity of the deposited on the soil surface radionuclides:

(3) $$\Delta {\dot{H}}_{\mathrm{estimated}}=\sum_{j=1}^{n}\left({\dot{H}}_{j}^{1Bq}\cdot A_{j}^{s}\left(t=t_{\mathrm{end}}\right)\right),\;\mu \mathrm{Sv}/h$$

where *j* is a radionuclide, and n is the number of deposited radionuclides.

The dose rate of γ-radiation created at the distance *R* from the soil surface (source) by a certain *j*-th radionuclide of unit activity is defined by constant value ${\dot{H}}_{j}^{1Bq}$ [17]. The quantity ${\dot{H}}_{j}^{1Bq}\left(R\right)$ is equal to the equivalent dose rate produced by the *j*-th radionuclide of unit activity at a certain distance *R* from an emitting object of arbitrary geometric shape; it can be calculated using the gamma constant of a radionuclide by equivalent dose rate (SGRDC) [17] from equations described in [18,19,20], and with GEANT4.

In this work, the dose coefficients for ${}^{214}$Pb and ${}^{214}$Bi were calculated using GEANT4 [21] at the height R=1 m from the ground surface for a disk source with a radius of 500 m, taking into account the lower threshold of γ-radiation registration by BDKG-03 detectors, equal to 50 keV. The standard set of physical processes QGSP_BIC_HP embedded in GEANT4 was used with some modification for our problem, similar to the example “extended/radioactivedecay/rdecay02” from the GEANT4 library. The statistics comprised 20 billion events for each individual calculation (radionuclide). The dose coefficients are as follows:

$$\begin{array}{c} {\dot{H}}_{\mathrm{Pb}-214}^{1Bq}=8.48\cdot 10^{-7},\left(\mu \mathrm{Sv}/h\right)/\left({\mathrm{Bq}/m}^{2}\right);\\ {\dot{H}}_{\mathrm{Bi}-214}^{1Bq}=4.86\cdot 10^{-6},\left(\mu \mathrm{Sv}/h\right)/\left({\mathrm{Bq}/m}^{2}\right). \end{array}$$

Next, we search for the unknown value $A_{j}^{s}\left(t=t_{\mathrm{end}}\right)$ by making the assumption that the activity of radon decay products ${}^{214}$Pb and ${}^{214}$Bi in clouds is negligible, or they have almost decayed during the motion of the cloud and they can be neglected. In this case, the activity of lead and bismuth deposited on the soil surface $A_{\mathrm{Pb}-214}^{s}\left(t\right)$ and $A_{\mathrm{Bi}-214}^{s}\left(t\right)$ is a function of time and is determined by their total activity in the atmosphere, the intensity and duration of precipitation, or the amount of precipitation.

It is practically impossible to measure the activity dynamics of ${}^{214}$Pb and ${}^{214}$Bi radon decay products precipitated on the ground surface or their activity at the moment of precipitation termination as well as the total activity of these radionuclides in the near ground atmosphere. In the equilibrium state, when the activities of radon and its decay products are equal, in the absence of rain, and when h→∞, $A_{\mathrm{Pb}-214}^{h}$ and $A_{\mathrm{Bi}-214}^{h}$ can be determined from the value of radon flux density from the ground surface q_Rn_, Bq m^−2^ s^−1^, from the simple equation as follows:

(4) $$A_{\mathrm{Rn}-222}^{h}\left(t=0\right)=A_{\mathrm{Po}-218}^{h}\left(t=0\right)=A_{\mathrm{Pb}-214}^{h}\left(t=0\right)=A_{\mathrm{Bi}-214}^{h}\left(t=0\right)=\frac{q_{\mathrm{Rn}}}{\lambda_{\mathrm{Rn}}}$$

where $\lambda_{\mathrm{Rn}}$ is radon radioactive decay constant ^222^Rn, s^−1^; $A_{\mathrm{Rn}-222}^{h}$, $A_{\mathrm{Po}-218}^{h}$, $A_{\mathrm{Pb}-214}^{h}$ and $A_{\mathrm{Bi}-214}^{h}$ are the integral values of volumetric activities of ${}^{222}$Rn and its short-lived decay products ${}^{218}$Po, ${}^{214}$Pb and ${}^{214}$Bi in an atmospheric column with a unit base and height *h*, Bq/m^2^.

During the period of precipitation, the integral values of the activity of radionuclides in a column of height *h* can be determined by solving the following system of equations:

(5) $$\left\{\begin{array}{c} \frac{dA_{\mathrm{Rn}}^{h}\left(t\right)}{dt}=q_{\mathrm{Rn}}-\lambda_{\mathrm{Rn}}\cdot A_{\mathrm{Rn}}^{h}\left(t\right),\\ \frac{dA_{\mathrm{Po}}^{h}\left(t\right)}{dt}=\lambda_{\mathrm{Po}}\cdot A_{\mathrm{Rn}}^{h}\left(t\right)-\left(\lambda_{\mathrm{Po}}+L\left(t\right)\right)\cdot A_{\mathrm{Po}}^{h}\left(t\right),\\ \frac{dA_{\mathrm{Pb}}^{h}\left(t\right)}{dt}=\lambda_{\mathrm{Pb}}\cdot A_{\mathrm{Po}}^{h}\left(t\right)-\left(\lambda_{\mathrm{Pb}}+L\left(t\right)\right)\cdot A_{\mathrm{Pb}}^{h}\left(t\right),\\ \frac{dA_{\mathrm{Bi}}^{h}\left(t\right)}{dt}=\lambda_{\mathrm{Bi}}\cdot A_{\mathrm{Pb}}^{h}\left(t\right)-\left(\lambda_{\mathrm{Bi}}+L\left(t\right)\right)\cdot A_{\mathrm{Bi}}^{h}\left(t\right), \end{array}\right.$$

where $\lambda_{\mathrm{Po}}$, $\lambda_{\mathrm{Pb}}$ and $\lambda_{\mathrm{Bi}}$ are the constants of the radioactive decay of isotopes ${}^{218}$Po, ${}^{214}$Pb, ${}^{214}$Bi, measured in s${}^{-1}$; $L\left(t\right)=I\left(t\right)\cdot k_{1}\cdot k_{2}$ is a function of the washout coefficient versus time, measured in s${}^{-1}$; where I(t) is the precipitation intensity function versus time; $k_{1}=10^{-5}$ (h/(mm·s)) is the absolute washout ability coefficient; and $k_{2}$ is relative washout ability coefficient.

The activity functions of radionuclides deposited on the soil surface by precipitation as a function of time can be determined from the expressions obtained by solving the Equations (5) and (6). The equations take into account such physical processes as radioactive decay of short-lived radon decay products in the atmosphere and on the soil surface during precipitation, as well as atmospheric clearance of radionuclides.

(6) $$\left\{\begin{array}{c} \frac{dA_{Po}^{s}\left(t\right)}{dt}=L\left(t\right)\cdot A_{Po}^{h}\left(t\right)-\lambda_{Po}\cdot A_{Po}^{s}\left(t\right),\\ \frac{dA_{Pb}^{s}\left(t\right)}{dt}=L\left(t\right)\cdot A_{Pb}^{h}\left(t\right)+\lambda_{Pb}\cdot A_{Po}^{s}\left(t\right)-\lambda_{Pb}\cdot A_{Pb}^{s}\left(t\right),\\ \frac{dA_{Bi}^{s}\left(t\right)}{dt}=L\left(t\right)\cdot A_{Bi}^{h}\left(t\right)+\lambda_{Bi}\cdot A_{Pb}^{s}\left(t\right)-\lambda_{Bi}\cdot A_{Bi}^{s}\left(t\right). \end{array}\right.$$

We make this complicated pathway much easier by representing $A_{\mathrm{Pb}-214}^{s}\left(t\right)$ and $A_{\mathrm{Bi}-214}^{s}\left(t\right)$ as follows:

$$\begin{array}{c} A_{\mathrm{Pb}-214}^{s}\left(t\right)=A_{\mathrm{Pb}-214}^{h}\left(t=0\right)-A_{\mathrm{Pb}-214}^{h}\left(t\right)=\frac{q_{\mathrm{Rn}}}{\lambda_{\mathrm{Rn}}}-A_{\mathrm{Pb}-214}^{h}\left(t\right),{\mathrm{Bq}/m}^{2};\\ A_{\mathrm{Bi}-214}^{s}\left(t\right)=A_{\mathrm{Bi}-214}^{h}\left(t=0\right)-A_{\mathrm{Bi}-214}^{h}\left(t\right)=\frac{q_{\mathrm{Rn}}}{\lambda_{\mathrm{Rn}}}-A_{\mathrm{Bi}-214}^{h}\left(t\right),{\mathrm{Bq}/m}^{2}; \end{array}$$

**Figure 4:**
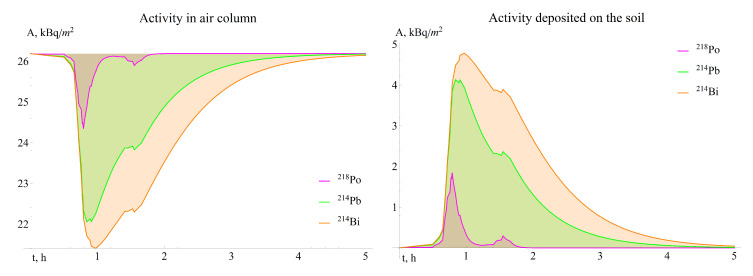
Dynamics of radon daughter decay products activity in the atmosphere and on the soil surface during the precipitation period on 11 June 2017.

Such a representation became possible after analyzing the dynamics of radon activity in the atmospheric column and on the ground surface during precipitation (Figure 4) obtained by solving Equations (5) and (6). It can be seen that the activities of radon deposited on the soil surface by precipitation daughter decay products change symmetrically together with the activities of these radionuclides in the atmosphere.

Then, we obtain the analytical solution only for system (5) of differential equations with constant coefficients and initial conditions (4). We obtained the solution (7) of equations of system (5) in the assumption that the intensity of precipitation is a constant value during one event, because by not having experimental data of the precipitation gauge in advance, it is impossible to assume the spectrum (dynamics) of intensity. As a result, one can determine $A_{\mathrm{Pb}-214}^{h}\left(t\right)$ and $A_{\mathrm{Bi}-214}^{h}\left(t\right)$ from Equation (7). For convenience in presenting the solution, we replace indexes ^222^Rn, ^218^Po, ${}^{214}$Pb and ${}^{214}$Bi with 1, 2, 3 and 4, respectively.

(7) $$\begin{array}{cc} & A_{3}^{h}\left(t\right)=\frac{qe^{-t(L+\lambda_{2})}\left(L\lambda_{2}e^{t(\lambda_{2}-\lambda_{3})}\left(L+\lambda_{2}\right)+\lambda_{2}\lambda_{3}e^{t(L+\lambda_{2})}\left(\lambda_{2}-\lambda_{3}\right)-L\lambda_{3}\left(L+\lambda_{3}\right)\right)}{\lambda_{1}\left(L+\lambda_{2}\right)\left(L+\lambda_{3}\right)\left(\lambda_{2}-\lambda_{3}\right)}\\ & X=-L\lambda_{2}\lambda_{3}e^{t(\lambda_{2}+\lambda_{3})}\left(L+\lambda_{2}\right)\left(\lambda_{2}-\lambda_{3}\right)\left(L+\lambda_{3}\right)\\ & Y=-\lambda_{2}\lambda_{3}\lambda_{4}e^{t(L+\lambda_{2}+\lambda_{3}+\lambda_{4})}\left(\lambda_{2}-\lambda_{3}\right)\left(\lambda_{2}-\lambda_{4}\right)\left(\lambda_{3}-\lambda_{4}\right)\\ & Z=L\lambda_{2}\lambda_{4}e^{t(\lambda_{2}+\lambda_{4})}\left(L+\lambda_{2}\right)\left(\lambda_{2}-\lambda_{4}\right)\left(L+\lambda_{4}\right)\\ & W=-L\lambda_{3}\lambda_{4}e^{t(\lambda_{3}+\lambda_{4})}\left(L+\lambda_{3}\right)\left(\lambda_{3}-\lambda_{4}\right)\left(L+\lambda_{4}\right)\\ & A_{4}^{h}\left(t\right)=\frac{qe^{-t(L+\lambda_{2}+\lambda_{3}+\lambda_{4})}\left(X+Y+Z+W\right)}{\lambda_{1}\left(L+\lambda_{2}\right)\left(L+\lambda_{3}\right)\left(L+\lambda_{4}\right)\left(\lambda_{2}-\lambda_{3}\right)\left(\lambda_{2}-\lambda_{4}\right)\left(-\lambda_{3}+\lambda_{4}\right)} \end{array}$$

where *L* is the coefficient of aerosol washout by precipitation, equal to $L=\bar{I}\cdot k_{1}\cdot k_{2}$, where $k_{1}$ is the coefficient of absolute washout ability of precipitation, equal to 36 m${}^{-1}$ (10${}^{-5}$ h/(mm·s)) [22]; $k_{2}$ is the coefficient of relative washout ability of precipitation, rel. units, equal to 1 for rain [22]; $\bar{I}$ is an average intensity of precipitation during the event, m/s.

Let us substitute solutions (7) into Equation (3) and rewrite initial equality (1) as follows:

(8) $$\Delta {\dot{H}}_{\mathrm{measured}}=\frac{q_{\mathrm{Rn}}}{\lambda_{\mathrm{Rn}}}\left({\dot{H}}_{\mathrm{Pb}-214}^{1Bq}+{\dot{H}}_{\mathrm{Bi}-214}^{1Bq}\right)-{\dot{H}}_{\mathrm{Pb}-214}^{1Bq}\cdot A_{3}^{h}\left(t_{\mathrm{end}}\right)-{\dot{H}}_{\mathrm{Bi}-214}^{1Bq}\cdot A_{4}^{h}\left(t_{\mathrm{end}}\right)$$

where $t_{end}$ is duration of precipitation, s.

We numerically solve Equation (8) with respect to *L*, provided that L>0, and obtain the event-average precipitation intensity $\bar{I}$, and multiplied by *t*_end_ we obtain the event-average precipitation $\bar{Q}$.

So our model (8) is called ^γ^R2P because it allows to turn data on γ-**R**adiation **To P**recipitation for estimating average quantity and intensity.

### 4.2. Correction for Radon Flux Density from the Ground Surface

**Figure 5:**
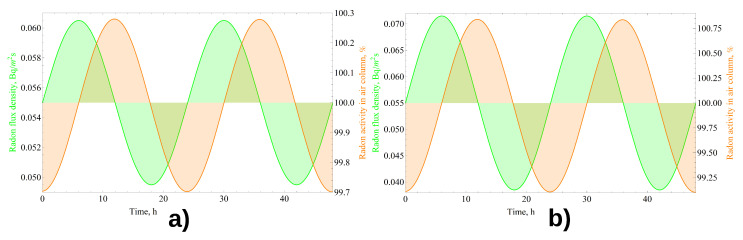
Dynamics of radon flux density from the soil surface and integrated radon activity in the atmosphere during 2 days at deviation of RFD from the average value: (**a**) by 10%; (**b**) by 30%.

As a rule, during the warm period of the year, radon flux density (RFD) experiences diurnal variations, which, depending on weather conditions, can change by 5–30 % or more [23], with a maximum at ≈6:00 a.m. and a minimum at ≈6:00 p.m. If we consider how the activity in the air column varies with this, we obtain a time shift in the onset of maxima and minima by 6 h (Figure 5). Radon activity dynamics in air column was simulated using the 1st equation of system (5), assuming $q\left(t\right)=q_{0}\cdot \left(1+A\cdot sin\left(\frac{2\pi }{T}\right)\right)$; here, *A* is the amplitude of variation, *T* is the period of variations, and $q_{0}$ is the average value of RFD. Variations of the RFD up to 30% (Figure 5b) result in insignificant changes, which can be neglected in the calculations. Nevertheless, if the RFD value changes many times, then for implementation of the GammaRain method, it is recommended to take RFD values measured 6 h before the beginning of rainfall. This will reduce the inaccuracy of the estimates of the intensity and the amount of precipitation.

With very heavy rainfall, when rainwater has no time to be absorbed by the soil, a “water layer” may occur for some time, blocking the radon output. However, this will not significantly affect the total activity of radon under the cloud since the half-life of ${}^{222}$Rn is 3.8 days, in addition, radon gas is not washed out of the atmosphere by precipitation. Let us consider as an example the case of an intense rain of 30 min duration, which presumably will reduce radon flux density to zero (which in reality is impossible, since radon diffuses even through water). If RFD = 55 mBq/(m${}^{2}$s) before heavy rain and then decreases sharply to zero, the total radon activity in the air column will decrease by less than 0.3% during 30 min of precipitation.

### 4.3. Reconstruction of the Rain Intensity Spectrum

**Figure 6:**
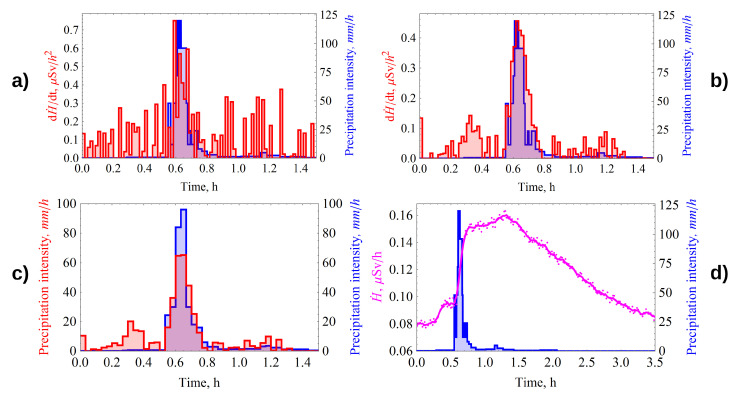
Dynamics of the measured precipitation intensity (blue) and time derivative of the measured dose rate (red) without smoothing (**a**), with smoothing by moving average (**b**), with averaging over 2 min (**c**), gamma dose rate (pink) (**d**).

The spectrum reconstruction is performed by the asserting equality of total precipitation amount between estimation of average (calculated using Task 1) and unknown one, which we have as time spectrum. So we know that $\frac{d\dot{H}\left(t\right)}{dt}∼I\left(t\right)\Rightarrow I\left(t\right)=c\cdot \frac{d\dot{H}\left(t\right)}{dt}$ and then we can integrate this equation to get precipitation amount. That is also true for average intensity, as the result $\int_{0}^{t_{end}}\frac{{\dot{H}}_{t}^{\prime }\left(t\right)}{\Delta t}dt\cdot c=\bar{I}$(average), finding constant in this equation allows us to use dose rate derivative for reconstruction of rain intensity spectrum. This principle is used for calculations and shown on Figure 6c and later.

## 5. Experimental Verification of the GammaRain Method

Next, we will discuss the implementation of the GammaRain method on several cases of rain.

Consider the rain that occurred on 25 June 2017 at 16:19 h, its duration was 42 min. The average 1-min intensity value changed to a maximum of 120 mm/h. The intensity spectrum is shown in blue in Figure 6a. The average measured value of radon flux density from the soil surface was 52 mBq/(m${}^{2}$s).

The dynamics of time derivative of gamma-radiation dose rate $\frac{d{\dot{H}}_{\mathrm{measured}}\left(t\right)}{\mathrm{dt}}$ calculated from the radiation monitoring data is shown in red. Figure 6a shows the spectrum of the derivative calculated from the original data measured at a sampling rate of 1 min. For the measurement conditions described in Section 2, the statistical error of the gamma monitoring data was 4–10%.

Due to the fact that the scatter of γ-background data (Figure 6d, pink) is quite large, we see in Figure 6a a discrepancy between the shape of the derivative spectrum (red) and the shape of the intensity spectrum (blue). When smoothing by “moving average” over the 5 values, we obtain an acceptable agreement of the spectra shapes of the $\frac{d{\dot{H}}_{\mathrm{measured}}\left(t\right)}{\mathrm{dt}}$ and $I_{\mathrm{measured}}\left(t\right)$ from onset of the intense phase of rain (Figure 6b).

The spectrum $I_{\mathrm{reconstructed}}\left(t\right)$ reconstructed by the GammaRain method (red) is shown in Figure 6c. The values of the calculated and reconstructed rainfall intensities are averaged over 2 min. The shapes of the calculated and original precipitation intensity spectra agree well, except for the initial section. This may be due to the error in intensity measurement using the shuttle gauge at the initial moment of rainfall.

Calculated by the ^γ^R2P model using Wolfram Mathematica, the average per-event rainfall intensity was 12.84 mm/h, and the average measured intensity was 11.7 mm/h.

Next consider the rainfall that occurred on 30 June 2017 at 16:57, which was 15 min in duration. The average value of intensity changed to a maximum of 168 mm/h in 1 min. The mean measured radon flux density from the ground surface was about 45 mBq/(m${}^{2}$s).

**Figure 7:**
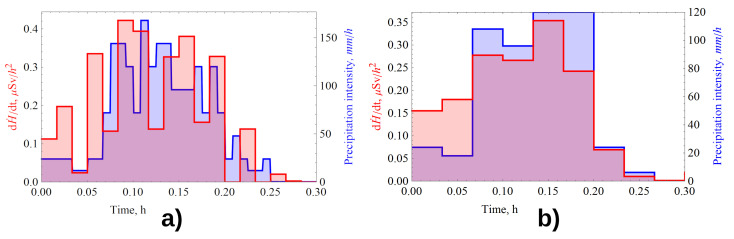
Dynamics of the intensity of precipitation on 30 June (blue) and the time derivative of the measured dose rate (red): (**a**) original data for 1 min; (**b**) averaging of 2 min.

Figure 7a shows the derivative $\frac{d{\dot{H}}_{\mathrm{measured}}\left(t\right)}{\mathrm{dt}}$ spectrum (red) calculated from the original gamma monitoring data measured at a sampling rate of 1 min, and the precipitation intensity (blue) averaged over 1 min. Figure 7b shows the derivative spectrum $\frac{d{\dot{H}}_{\mathrm{measured}}\left(t\right)}{\mathrm{dt}}$ and $I_{\mathrm{measured}}\left(t\right)$ both averaged over 2 min. The time-varying derivative of the measured dose rate agrees well with the dynamics of the measured precipitation intensity.

The average value of the measured intensity for the case was 70.4 mm/h, while the one calculated using the ^γ^R2P model was 70.6 mm/h.

**Figure 8:**
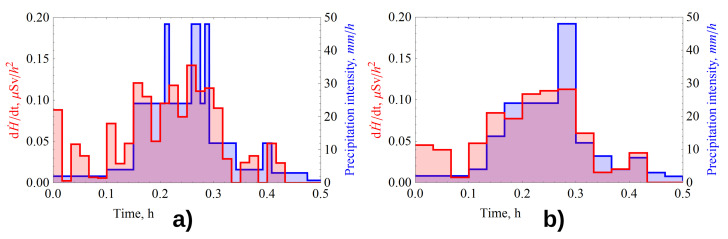
Dynamics of the time derivative of the measured dose rate (red) and intensity of precipitation on 11 June (blue) with averaging: (**a**) 1 min; (**b**) 2 min.

Figure 8 shows the results of comparing the shapes of the measured intensity and time derivative dose rate spectra for the rain that occurred on 11 June 2017 at 18:15. The rain duration was 30 min, the average 1 min intensity value changed to a maximum of 48 mm/h The average measured radon flux density from the ground surface was 47 mBq/(m${}^{2}$s).

The shapes of the derivative and intensity spectrum are satisfactorily similar at averaging of 1 and 2 min. The average value of the measured intensity for the case was 11.6 mm/h, and the value calculated by the model ${}^{\gamma }$R2P was 11.0 mm/h.

**Figure 9:**
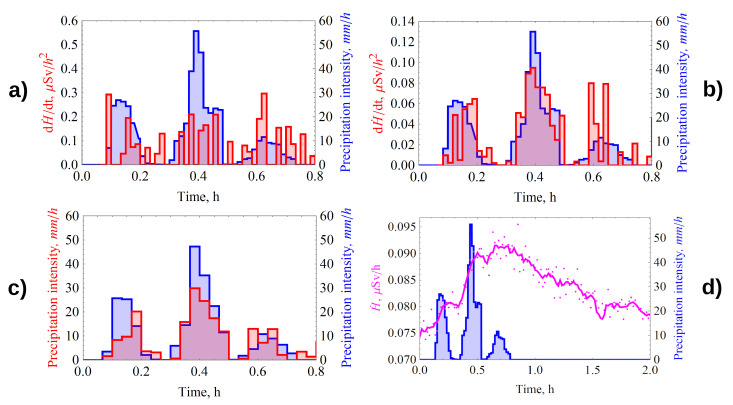
Dynamics of the measured precipitation intensity (blue) and time derivative of the measured dose rate (red) without smoothing (**a**), with smoothing by moving average (**b**), with averaging over 2 min (**c**), gamma radiation dose rate (pink) (**d**).

Let us also give the results of the analysis of a rain of complex shape, having 3 intense phases in one event, with a spectrum resembling a “corona” in shape (Figure 9). This rain occurred on 30 July 2017 at 11:53, its duration was 48 min. The average 1-min intensity value changed to a maximum of 55.7 mm/h, the intensity spectrum is shown in blue in Figure 9. Average measured value of radon flux density from the soil surface averaged 36 mBq/(m${}^{2}$s).

Dynamics of time derivative of gamma-radiation dose rate calculated from gamma-background monitoring data are shown in red. In spite of the larger scatter of gamma-background data (Figure 9d, pink, dots), compared to the rain in Figure 6d, the derivative in Figure 9b follows the rain spectrum shape quite well.

The spectrum reconstructed using the GammaRain method is shown in Figure 6c (red). In the absolute value, the reconstructed intensity values are slightly underestimated compared to the measured values.

The average measured intensity per case was 10.1 mm/h, while the calculated one was 7.8 mm/h.

Analysis of the results of rainfall spectra reconstruction by gamma-background as well as estimation of average per event precipitation intensities showed the following:

(1) The developed method gives a good matching of the reconstructed and measured intensity spectra shapes;
(2) The event-averaged rainfall intensity calculated using the ^γ^R2P model agrees perfectly with the measured value for rainfall with the form of type I spectrum, and for complex spectra, the error may reach 25%.

Reducing the sampling rate of the radiation monitoring data will increase the error in the estimates of precipitation characteristics. In order to obtain higher temporal resolution while keeping the error at the same level, or smaller, it is necessary to select more sensitive sensors (for example, larger size of NaI(Tl) scintillator sensitive volume). This will allow in the dynamics of γ-background to distinguish more precisely the areas with different rate of rise (angle of slope of derivative).

## 6. Conclusions

Analysis of the results of long-term experiment on investigation of features of atmospheric γ-background response to liquid atmospheric precipitation allowed to establish that the time derivative of the dose rate $\frac{d\dot{H}\left(t\right)}{\mathrm{dt}}$ follows well the intensity spectrum of liquid atmospheric precipitation. Analysis of the γ-background response to rains of different intensity and duration allowed the following:

- To state that it has rained;
- To formulate distinctive features by which we can determine the time of the beginning and end of precipitation, changes in the intensity of precipitation and the number of single (individual) events that form one burst in the γ-background;
- To determine the average intensity (quantity) of rainfall;
- To reconstruct the spectrum of rain intensity.

The ^γ^R2P model was developed to estimate the average values of intensity and quantity of rainfall per single event, using the experimental data on the dynamics of the γ-radiation dose rate. This model takes into account radioactive decay products of radon decay in the atmosphere and on the soil surface during precipitation, as well as atmospheric clearance from radionuclides.

Experimental verification of the developed GammaRain method of rain intensity spectrum reconstruction using the measured γ-background showed satisfactory agreement between the restored and measured rain intensity spectra.

## Data Availability

The data presented in this study are available on request from the corresponding author. The data are not publicly available due to privacy reasons.

## Abbreviations

The following abbreviations are used in this manuscript:

Ra	radium
Rn	radon
Po	polonium
Pb	plumbum
Bi	bismuth
RF	Russian Federation
TPU	Tomsk Polytechnic University
IKIR FEB RAS	Institute of Cosmophysical Research and Radio Wave Propagation,
	Far Eastern Branch of the Russian Academy of Sciences
IMCES SB RAS	Institute of Monitoring of Climatic and Ecological Systems
	of Siberian Branch of the Russian Academy of Sciences
